# Disease Interactions in a Shared Host Plant: Effects of Pre-Existing Viral Infection on Cucurbit Plant Defense Responses and Resistance to Bacterial Wilt Disease 

**DOI:** 10.1371/journal.pone.0077393

**Published:** 2013-10-14

**Authors:** Lori R. Shapiro, Lucie Salvaudon, Kerry E. Mauck, Hannier Pulido, Consuelo M. De Moraes, Andrew G. Stephenson, Mark C. Mescher

**Affiliations:** 1 Department of Entomology, Pennsylvania State University, University Park, Pennsylvania, United States of America; 2 Laboratoire Ecologie, Systématique et Evolution, Université Paris Sud, Orsay, France; 3 Department of Biology, Pennsylvania State University, University Park, Pennsylvania, United States of America; 4 Center for Infectious Disease Dynamics, University Park, Pennsylvania, United States of America; 5 Department of Environmental Systems Science, Eidgenössische Technische Hochschule Zürich, Zürich, Switzerland; French National Institute for Agricultural Research (INRA), France

## Abstract

Both biotic and abiotic stressors can elicit broad-spectrum plant resistance against subsequent pathogen challenges. However, we currently have little understanding of how such effects influence broader aspects of disease ecology and epidemiology in natural environments where plants interact with multiple antagonists simultaneously. In previous work, we have shown that healthy wild gourd plants (*Cucurbita pepo* ssp. *texana*) contract a fatal bacterial wilt infection (caused by *Erwinia tracheiphila*) at significantly higher rates than plants infected with Zucchini yellow mosaic virus (ZYMV). We recently reported evidence that this pattern is explained, at least in part, by reduced visitation of ZYMV-infected plants by the cucumber beetle vectors of *E. tracheiphila*. Here we examine whether ZYMV-infection may also directly elicit plant resistance to subsequent *E. tracheiphila* infection. In laboratory studies, we assayed the induction of key phytohormones (SA and JA) in single and mixed infections of these pathogens, as well as in response to the feeding of *A. vittatum* cucumber beetles on healthy and infected plants. We also tracked the incidence and progression of wilt disease symptoms in plants with prior ZYMV infections. Our results indicate that ZYMV-infection slightly delays the progression of wilt symptoms, but does not significantly reduce *E. tracheiphila* infection success. This observation supports the hypothesis that reduced rates of wilt disease in ZYMV-infected plants reflect reduced visitation by beetle vectors. We also documented consistently strong SA responses to ZYMV infection, but limited responses to *E. tracheiphila* in the absence of ZYMV, suggesting that the latter pathogen may effectively evade or suppress plant defenses, although we observed no evidence of antagonistic cross-talk between SA and JA signaling pathways. We did, however, document effects of *E. tracheiphila* on induced responses to herbivory that may influence host-plant quality for (and hence pathogen acquisition by) cucumber beetles.

## Introduction

Research conducted over the past several decades has shown the signaling networks and molecular mechanisms of induced plant response to biotic and abiotic stressors to be highly complex and tightly regulated; but our understanding of how such responses function in complex ecological environments where plants simultaneously interact with multiple antagonists remains limited, particularly for non-model species [1]. In this study, we examine the simultaneous interactions of a wild gourd (*Cucurbita pepo* ssp. *texana*) with disparate but frequently co-occurring microbial pathogens and an insect herbivore. Specifically, we explore constitutive and induced plant responses to single and mixed infections of Zucchini yellow mosaic virus (ZYMV) and *Erwinia tracheiphila* (the causal agent of bacterial wilt disease) and the further influence on these responses of feeding by a key specialist herbivore—the striped cucumber beetle, *Acalymma vittatum*—that vectors the bacterial pathogen [2]. This system is well suited to examining plant defense response in the context of complex ecological interactions, as we have extensive knowledge of co-infection dynamics for these pathogens and of *A. vittatum* host preferences under field conditions. 

Both ZYMV and *E. tracheiphila* are endemic in populations of wild gourds planted in central Pennsylvania (our study location). Yet, previous work indicates that these pathogens co-infect individual host plants less frequently than would be expected by chance, and specifically that plants with prior ZYMV infections subsequently contract *E. tracheiphila* infections—which are invariably lethal once symptoms appear—at a greatly reduced rate compared to healthy plants [3,4]. Recent evidence suggests that this pattern is mediated, at least in part, by reduced exposure of ZYMV-infected plants to the beetle vectors of *E. tracheiphila*: ZYMV-infected plants produce fewer flowers than healthy plants [3,5], and flowers that are produced on these plants exhibit significantly attenuated production of floral odors that are key attractants for cucumber beetles, which aggregate in cucurbit flowers to mate and feed [6,7]. Consequently, *A. vittatum* individuals exhibit a preference for the odors of healthy vs. virus-infected flowers in laboratory assays and are also much more abundant in healthy than infected flowers in the field [7]. This is expected to influence rates of *E. tracheiphila* infection, as we have shown that this pathogen is efficiently transmitted through floral nectaries [8] and that the incidence of wilt disease in populations of *C. pepo* ssp. *texana* is strongly influenced by the presence and abundance of flowers [3,9].

Despite the likelihood that reduced exposure of ZYMV-infected plants to the beetle vectors of *E. tracheiphila* contributes to the relative infrequency of co-infections by these two pathogens, direct effects of virus infections on host plant physiology may also be important, and have not previously been explored. The current study therefore documents *in planta* changes in key signaling molecules mediating plant defense responses following infection by ZYMV and *E. tracheiphila* and specifically explores whether infection by the viral pathogen induces systemic acquired resistance (SAR) that reduces plant susceptibility to bacterial wilt disease. In addition to providing novel insights into the interactions between these pathogens, this study elucidates the pathogen-induced defense responses of the wild gourd *C. pepo* ssp. *texana*, which may be of significant interest in relation to previous work on cultivated cucumber (*Cucumis sativa*, Cucurbitaceae). *Cucurbita* and *Cucumis* share similar suites of microbial and insect antagonists, including the pathogens addressed in this study and their herbivorous insect vectors—*E. tracheiphila* is vectored exclusively by specialist Diabroticite cucumber beetles (Coleoptera: Chrysomelidae: Luperini) [2], while ZYMV, an emerging viral pathogen of cucurbits worldwide, is transmitted in a non-persistent manner by several generalist aphid species [10]. While significant research has documented the signaling pathways mediating induced plant-defense responses in cultivated cucumber (as discussed below), little is currently known about how such responses may influence broader patterns of disease ecology and epidemiology in natural plant populations where multiple pathogens frequently co-occur [1].

Cultivated cucumber was an important model for early work on the regulation of plant defense responses, leading to the identification of salicylic acid (SA) as a mobile signal responsible for SAR [11] and elucidation of its apparent role in regulating the induction of pathogenesis-related (PR) proteins following plant inoculation with various bacterial, viral, and fungal pathogens [12]. Furthermore, SA-associated induced resistance in cucumber has been shown to be nonspecific: broad spectrum resistance to subsequent pathogen challenge can be induced by abiotic stressors like phosphates [13] as well as by biotic antagonists such as Tobacco necrosis virus (TNV) or the fungal pathogen *Colletotrichum lagenarium* (reviewed in [14]). 

SA is now generally recognized to be the primary phytohormone regulating induced pathogen resistance in plants, particularly against biotrophic pathogens like viruses, as well as biotrophic and hemibiotrophic bacteria [15,16] and is known to mediate broad-spectrum SAR, cell-wall fortification, and the accumulation of PR proteins [17,18]. However, other signaling molecules also mediate induced plant defenses, including cis-jasmonic acid (JA), which plays important roles in defense signaling [19,20] in addition to being involved in regulating plant growth and senescence [21]. JA has been most extensively studied in the context of plant responses to chewing herbivores, and JA-mediated anti-herbivore defenses include induction of toxic secondary metabolites, proteinase inhibitors, and insect growth suppressors [19,22]. However, JA has also been proposed to play a role in mediating defenses against some necrotrophic pathogens [15,23]. Furthermore, the JA and SA pathways have been shown to interact antagonistically in some systems [24,25], although so-called “cross talk” between these pathways has been documented in a relatively small number of model organisms and crop species and may not be universal [25].

To gain insight into the defense responses of *C. pepo* ssp. *texana* and their potential implications for the simultaneous interaction of this wild plant with multiple antagonists, we examined (i) changes in the levels of the key defense signaling molecules SA and JA elicited by *E. tracheiphila* and ZYMV, individually and in co-infection; (ii) the effects of prior infection by ZYMV on subsequent rates of infection by *E. tracheiphila*, as well as the timing and intensity of wilt-disease onset and progression; and (iii) the effects of prior infection by each pathogen on plant defense induction following feeding by *A. vittatum*.

## Materials and Methods

### Plants, pathogens, and herbivores

The wild gourd *Cucurbita pepo* spp. *texana* is thought to be the wild progenitor of cultivated squash varieties [26]. All experiments employed *C. pepo* spp. *texana* seeds from wild maternal families originally collected in Texas and subsequently grown yearly at the Penn State University Research Farm in Rock Springs, PA. Both ZYMV and *E. tracheiphila* occur naturally in these plant populations. (The incidence of these and other pathogens in wild populations of *C. pepo* spp. *texana* in its native range, which extends from the southern US through Mesoamerica, is not well characterized). After germination, seedlings were transplanted in pots with compost soil supplemented with 3g of Osmocote slow-release fertilizer (NPK:14-14-14) and micronutrients. All experiments were performed in climate-controlled chambers under incandescent and fluorescent lights (25°C, 16:8 light:dark photoperiod). 

**Figure 1:**
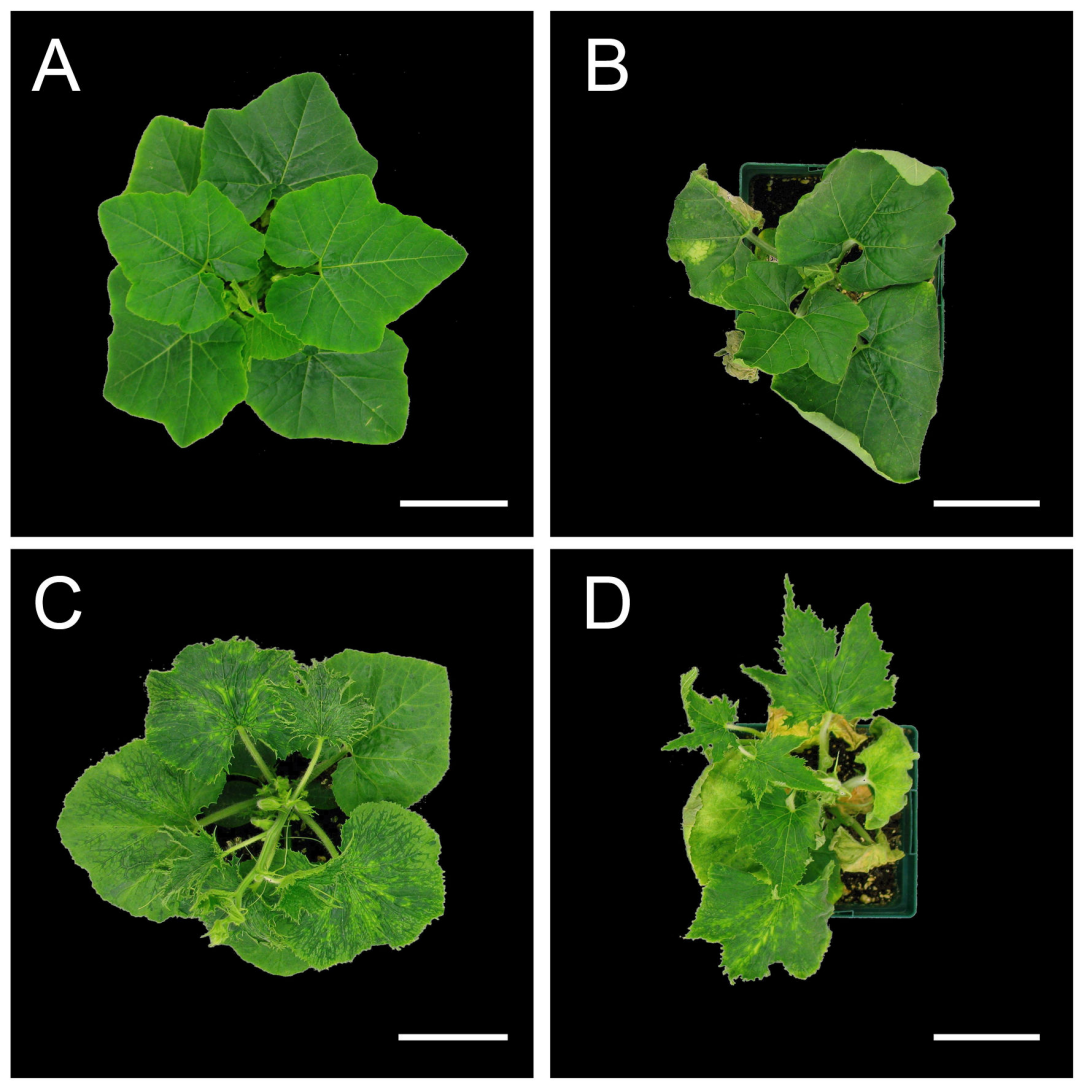
Symptoms of *C. pepo* ssp. *texana* plants infected with ZYMV and *E. tracheiphila*. (A) Healthy plant mock-inoculated for both viral and bacterial diseases; (B) Plant with *E. tracheiphila* wilt symptoms; (C) ZYMV-infected plant; (D) ZYMV-infected plant co-infected with *E. tracheiphila* and showing both virus and bacterial wilt symptoms.

ZYMV is single stranded, positive sense, RNA virus transmitted by a number of aphid species in a non-persistent manner [27,28]. A strain of ZYMV, originally sampled from a squash field in 2009, was maintained as a static stock culture at -80°C and used for all virus-inoculation experiments. Virus-infected plants typically exhibited mosaic symptoms, leaf deformations, and reduced growth (Figure 1A,C), which developed over the course of one to two weeks following inoculations (described below).

*E. tracheiphila* Smith (Enterobacteriaceae) is the causative agent of cucurbit bacterial wilt disease. It is acquired by spotted and striped cucumber beetles (*Diabrotica undecimpunctata* and *A. vittatum*, respectively) when they feed on wilting, symptomatic leaves and can be transmitted when infected frass falls onto feeding-damaged plant tissues or floral nectaries [7,8]. A local field isolate of *E. tracheiphila* was maintained on nutrient peptone agar at 27°C for 3 days before plant inoculations. In vitro-cultured bacteria, or infectious frass from beetles exposed to diseased plants, were injected into plants (protocols below). Successful bacterial establishment led to wilt symptoms on the inoculated leaf within a few days, followed by leaf necrosis, wilt of other leaves and branches, and eventually death of the plant (Figure 1B,D).

Striped cucumber beetles (*A. vittatum*) were originally collected from a local field population in Rock Springs, PA. A colony was propagated by keeping field collected beetles in 1ft x 1 ft Bioquip cages and feeding the colony leaves and flowers of *Cucurbita pepo* cv. ‘Raven’ 3 times per week. Moistened potting soil was provided as oviposition substrate. Soil (with eggs deposited) was transferred weekly to a new 14L plastic bin and supplied with sprouted *C. pepo* cv. ‘Raven’ seedlings, on which rootworm larvae fed until pupation. Newly eclosed adults were immediately transferred to a new Bioquip mesh cage.

### Hormone induction in response to pathogen infections and herbivory

To examine constitutive and induced levels of defense-related phytohormones, 83 *C. pepo* spp. *texana* seedlings at the two true leaves stage were randomly divided into three virus treatment groups (ZYMV-inoculated, mock-inoculated controls, and untouched controls). Mechanical inoculation of plants with ZYMV was accomplished by dusting leaves with carborundum and using cotton swabs to apply an inoculum comprising infected tissue ground in 0.1M potassium-phosphate buffer (the equivalent of a 4cm diameter leaf disc for 10 mL of buffer). Mock inoculations employed the same protocol but with clean buffer only. Infected plants exhibited initial virus symptoms within 5 days of inoculation. Seven days after virus or mock inoculation, when infected seedlings uniformly showed virus symptoms, half of the plants in each treatment group (selected at random) were inoculated with a virulent field isolate of *E. tracheiphila*. (In all experiments, we explored only co-infections involving plants initially infected with the viral pathogen and subsequently exposed to the bacterial pathogen because we are explicitly interested in understanding the reduced incidence of wilt disease in virus-infected plants and because, under field conditions, bacterial wilt infections are invariably lethal within several days). Bacterial inoculations were performed by scraping all bacteria from a single plate into 10mL of tap water in a beaker, mixing gently until cells were homogeneously dispersed, then applying 10μl of the bacterial suspension to a small break on the petiole of the newest fully expanded leaf. The other half of the plants within each of the virus treatments received the same procedure, except that clean water alone was used (a second mock inoculation for the bacterial treatment). 

At 48 hours following the bacterial and mock-bacterial inoculations, inoculated leaves were quickly harvested, weighed (0.1-0.2g range), and flash frozen in liquid nitrogen. Hormone extraction procedures were modified from those of Schmelz et al. [29,30]. Briefly, frozen tissue was ground with stainless steel beads in a Genogrinder under liquid nitrogen. While on ice, the ground tissue samples received 100ng of internal, isotopic standards for JA and SA (dH-JA and ^2^H_6_-SA) as well as 400μL of an aqueous, weakly acidic buffer solution to complete cell disruption. After mixing, each sample received 1mL of dichloromethane and was then mixed and centrifuged for 1 minute at 11,000xg. The dichloromethane layer, now containing the compounds of interest and the internal standards, was removed to a glass vial and dried under house air for 10-15 minutes. The residue was reconstituted in a mixture of 1:9 diethyl ether:methanol, and carboxylic acids within the solution (e.g., JA, SA) were derivatized to methyl esters using Trimethylsilydizomethane (Sigma-Aldrich, St. Louis, MO). After the reaction was stopped, the remaining solvent was allowed to evaporate, and the residue containing the methyl esters was heated for 2 min at 200°C to volatilize the esters. During heating, air was sampled from the vial by pulling through an adsorbent trap (30mg Super-Q) at a rate of 1L of air per minute. Super-Q traps were eluted with 150μl dichloromethane and the eluate was analyzed by GC-MS with isobutane chemical ionization and select monitoring of phytohormone ions described in [30]. Final concentrations of free phytohormones (cis-JA and SA) were calculated relative to recovery of the internal standards corrected by the original weight of the sample. Data were analyzed with treatment as the independent variable and phytohormone amounts (ln-transformed for normality) as the dependent variable in SAS PROC GLM [31] with the model statement ln_Hormone = Disease + *E. tracheiphila* inoculation + Disease*E. tracheiphila inoculation. 

To explore whether bacterial or viral infection affects induced responses of *C. pepo* ssp. *texana* to herbivory by *A. vittatum*, 68 seedlings were assigned for inoculation either as mock-inoculated controls, ZYMV-inoculated, or inoculated with *E. tracheiphila* that had been grown on nutrient peptone. ZYMV inoculations and mock-virus inoculations occurred when the seedlings had two true leaves, and *E. tracheiphila* inoculations were performed when plants had 4 true leaves so that ZYMV-infected plants and *E. tracheiphila* infected plants would show symptoms concurrently (all inoculations and mock inoculations were otherwise as above). Five days after *E. tracheiphila* inoculations when all infected plants showed either ZYMV or *E. tracheiphila* symptoms, half of each inoculation group were randomly subjected to herbivory from one striped cucumber beetle confined to the newest fully expanded leaf (with a clip cage, covering ~6 cm^2^ of leaf area) for 15 hours (overnight). After the insects were removed, 0.1-0.2 g of leaf tissue adjacent to the site of feeding damage (or from the corresponding leaves of plants not subjected to herbivory) was harvested, weighed, snap-frozen, and processed for hormone analysis (as above). Raw values (ng/g fresh weight) were ln transformed to meet assumptions of normality and analyzed with SAS PROC GLM [31] with the model statement ln_Hormone = Disease + Herbivory + Disease*Herbivory.

### Plant susceptibilty to *E. tracheiphila* infection

To explore potential differences in wilt susceptibility between healthy and ZYMV-infected plants at field-relevant levels of *E. tracheiphila* exposure, 125 wild gourd seedlings were randomly mock-inoculated or inoculated with ZYMV (as above). Seven days after viral or mock-inoculations, when the seedlings uniformly showed virus symptoms, all plants were inoculated with a homogenate of *A. vittatum* beetle frass containing *E. tracheiphila* (to mimic natural exposure). To accomplish these frass inoculations, 125 lab-reared *A. vittatum* were allowed to feed on symptomatic, *E. tracheiphila*-infected wild gourd plants for 24 hours, then groups of 10-12 beetles were immediately placed in petri dishes with a clean cucumber leaf. After 2 hours, all the frass from all petri dishes was collected and pooled in 1mL of tap water within a 1.7ml Eppendorf tube. The tube was inverted several times to disperse the frass evenly. A small break was made at the base of the petiole of a fully expanded leaf and 10μl of the frass homogenate was applied at the site of leaf breakage. The onset (first symptomatic leaf) and progression of wilt disease symptoms was recorded over time, and proportions of wilting, *E. tracheiphila*-infected plants in each treatment were compared with a χ^2^ test of two proportions in R [32].

Differences in wilt symptom development between healthy and ZYMV-infected plants at higher levels of *E. tracheiphila* exposure were also examined. For these experiments, 150 seedlings were randomly mock-inoculated, inoculated with ZYMV (as above), or left untouched. All plants were then inoculated, seven days later, with a homogenate of *E. tracheiphila*-infected beetle frass augmented with *in vitro*-grown bacterial colonies (protocols otherwise as above). The addition of cultured bacteria to the solution enabled us to deliver a higher dose of bacteria, while still exposing plants to any chemicals in beetle frass (e.g., phytohormones, plant secondary metabolites, or insect-derived elicitors) that might themselves influence plant defense responses. All but one plant developed wilt symptoms within 17 days after inoculation. As in the previous experiment, the onset and progression of wilt symptoms were recorded over time. The number of days until wilt symptoms appeared was ln transformed and analyzed with a one-way ANOVA in SAS Proc GLM for differences in time until wilt symptoms appeared. Two plants apparently recovered from the disease and were excluded from the whole plant wilting analysis. 

## Results

### Induction of hormones in response to pathogen infections and herbivory

**Figure 2:**
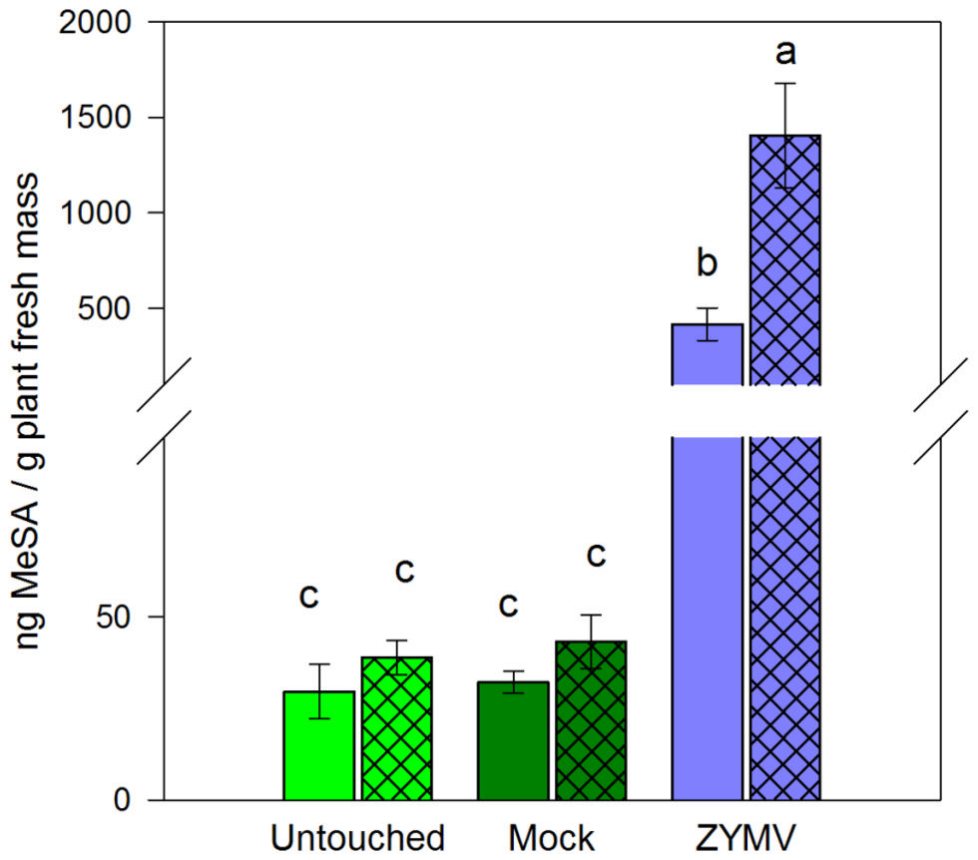
Salicylic acid responses of healthy and ZYMV-infected plants. Mean salicylic acid (+/- SE) measured 48hr after *E. tracheiphila* (or mock) inoculation. Untouched = healthy controls; Mock = mock virus inoculation; ZYMV = virus-infected. Open bars designate uninfected plants subjected to mock-inoculation with *E. tracheiphila*; hatched bars designate plants inoculated with *E. tracheiphila*. Letters indicate significant differences (*P* < 0.05 in a Tukey-Kramer test). Sample sizes (*E. tracheiphila* free : *E. tracheiphila*-infected): Untouched (14 : 14); Mock (14 : 13); ZYMV (15 : 12).

Leaves on ZYMV-infected wild gourd seedlings exhibited significantly higher levels of SA, both before and after subsequent infection with *E. tracheiphila*, compared to healthy (mock-inoculated and untouched control) plants (Figure 2, Table 1). Interestingly, co-infection by *E. tracheiphila* elicited significantly higher levels of SA in plants with prior ZYMV infections, but *E. tracheiphila* alone did not appear to induce an SA response in untouched or mock-virus-inoculated control plants (Figure 2, Table 1). Cis-JA was not detected in any of the pathogen-only treatments. 

**Table 1:** ANOVA table for SA levels (ln transformed) in response to virus treatments (Untouched, Mock or ZYMV), *E. tracheiphila* inoculation, and their interaction.

Source of Variation	Df	SS	MS	*F* ratio	*P*
Virus treatment	2	146.8	73.4	198.13	< 0.0001
*E. tracheiphila* inoculation	1	5.5	5.5	14.93	< 0.0001
Virus treatment x *E. tracheiphila*	2	7.0	3.5	9.40	0.0055
Error	76	28.2	0.37		
Corrected total	81	187.5			

**Figure 3:**
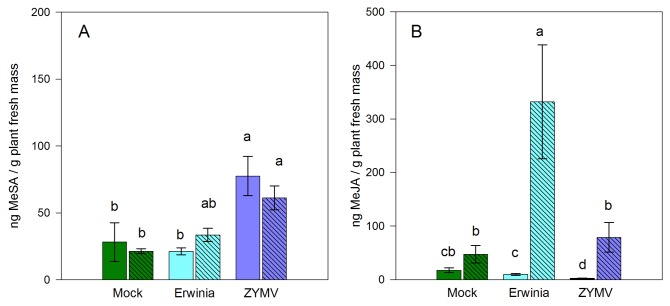
Phytohormone responses of healthy, *E. tracheiphila*-inoculated, and ZYMV-infected plants to cucumber beetle herbivory. Levels of the phytohormones SA and JA measured 15h after exposure to cucumber beetles. (A) mean salicylic acid production ± SE; (B) mean cis-JA ± SE. Mock = mock virus inoculation; *Erwinia* = inoculation with *E. tracheiphila*; ZYMV = inoculation with ZYMV. Open bars designate the undamaged treatment; Striped bars designate beetle-herbivory treatments. Letters indicate significant differences (*P* < 0.05 in a Tukey-Kramer test). Sample sizes (without herbivory : herbivory): Mock (13 : 12); *Erwinia* (12 : 10); ZYMV (13 : 13).

Significantly stronger SA responses to ZYMV compared to healthy plants or those infected by *E. tracheiphila* alone were again evident in the herbivory experiment (Figure 3A, Table 2) (Contrast “ZYMV” vs, “*E. tracheiphila*” and “mock” disease treatments *F*_1,66_= 39.04, *P* value < 0.0001), but feeding by *A. vittatum* did not appear to influence SA levels in any of the treatments. In contrast, cis-JA was significantly induced by beetle feeding in all treatments, and the JA response was particularly high in plants infected with *E. tracheiphila* compared to ZYMV-infected and healthy plants (Figure 3B, Table 3). 

**Table 2:** ANOVA table for SA levels (ln transformed) in response to disease treatment (Mock, ZYMV or *E. tracheiphila*), beetle herbivory, and their interaction.

Source of Variation	Df	SS	MS	*F* ratio	*P*
Disease	2	19.9	10.0	21.50	< 0.0001
Herbivory	1	0.8	0.8	1.70	0.1970
Disease x Herbivory	2	1.1	0.5	1.14	0.3261
Error	66	30.6	0.5		
Total	71	52.5			

**Table 3:** ANOVA table for cis-JA levels (ln transformed) in response to disease treatment (Mock, ZYMV or *E. tracheiphila*), beetle herbivory, and their interaction.

Source of Variation	Df	SS	MS	*F* ratio	*P*
Disease	2	21.1	10.5	12.53	< 0.001
Herbivory	1	86.0	86.0	102.32	< 0.001
Disease x Herbivory	2	14.6	7.3	8.7	0.0004
Error	67	56.3	0.8		
Total	72	171.8			

### Susceptibility to *E. tracheiphila*

**Figure 4:**
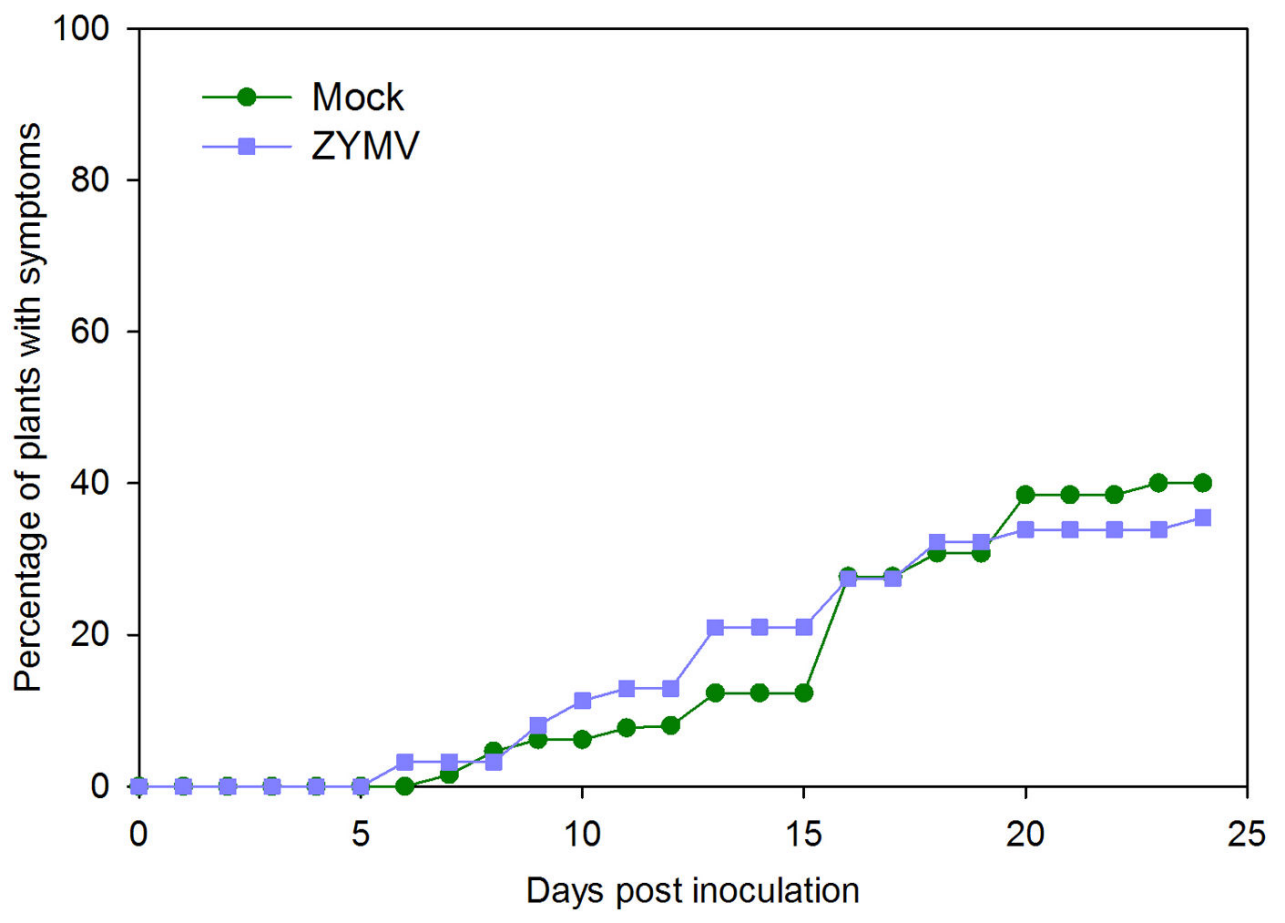
*E. tracheiphila* disease progression in healthy and ZYMV-infected plants after low-dose inoculations. Disease progression over 26 days for 62 healthy (mock-inoculated) plants, and 65 ZYMV-infected plants following artificial inoculation with infectious cucumber beetle frass.

In the inoculation experiment using only infectious beetle frass, no significant difference in the overall rate of *E. tracheiphila* infection success on ZYMV-infected plants relative to mock-inoculated (healthy) plants was observed (Figure 4). The first wilt symptoms appeared on ZYMV-infected plants one day later than on mock-infected plants (7 days vs. 6 days post *E. tracheiphila* inoculation), but no significant difference was observed in the percentage of plants contracting wilt disease, 35% vs. 40% for mock-inoculated and ZYMV-infected plants, respectively, at the conclusion of the experiment (χ^2^ = 0.1167; *P* = 0.733). 

**Figure 5:**
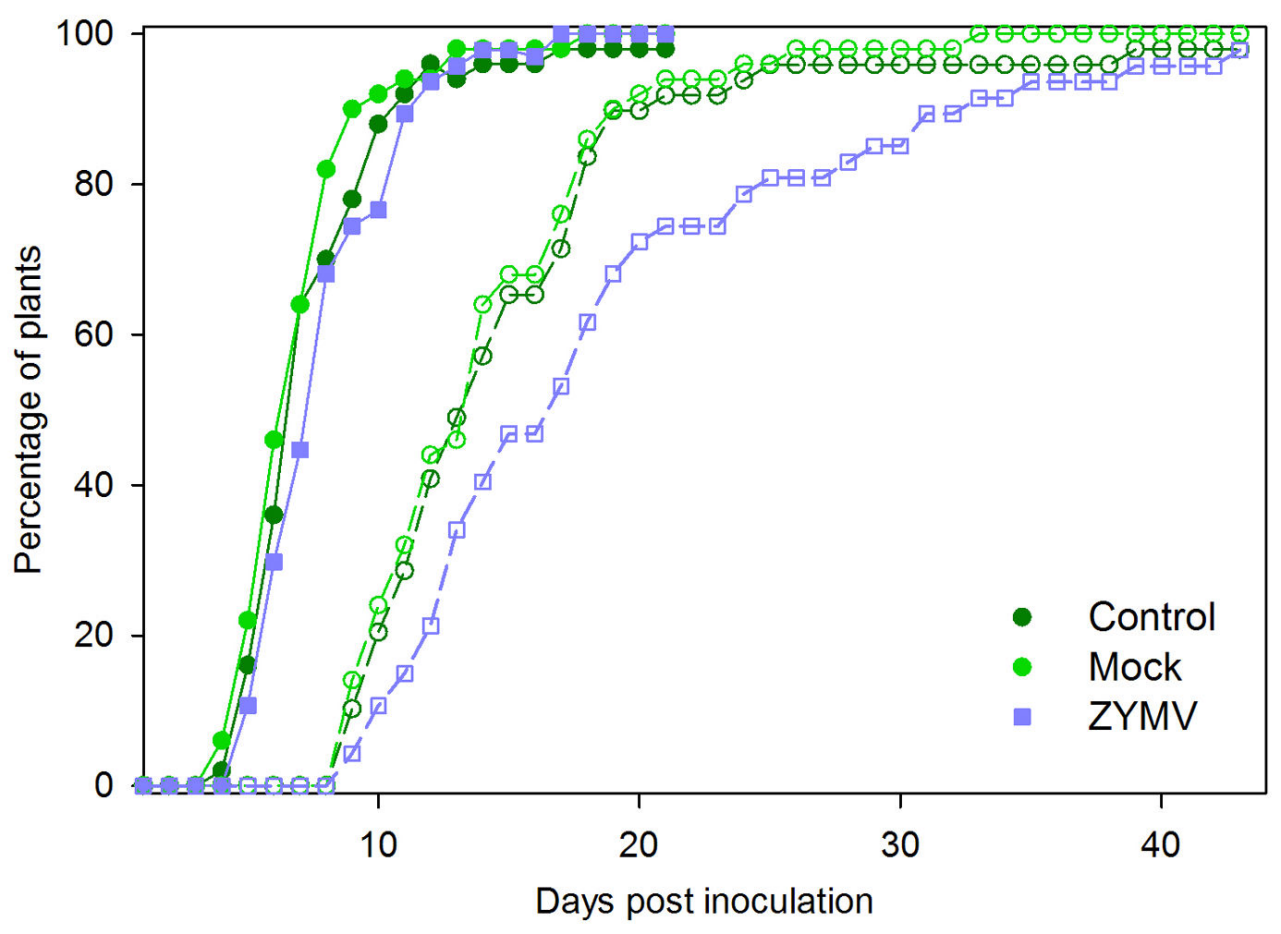
*E. tracheiphila* disease progression in healthy and ZYMV-infected plants after high-dose inoculations. Disease progression over 45 days for untouched controls (n=49), mock-virus-inoculated (n = 50), and ZYMV-infected (n = 47) plants following inoculation with a homogenate of infectious cucumber-beetle frass supplemented with *in* *vitro* cultured *E. tracheiphila*. Lines with closed symbols track the initial onset of wilt symptoms; lines featuring open symbols track complete plant collapse due to wilting.

**Figure 6:**
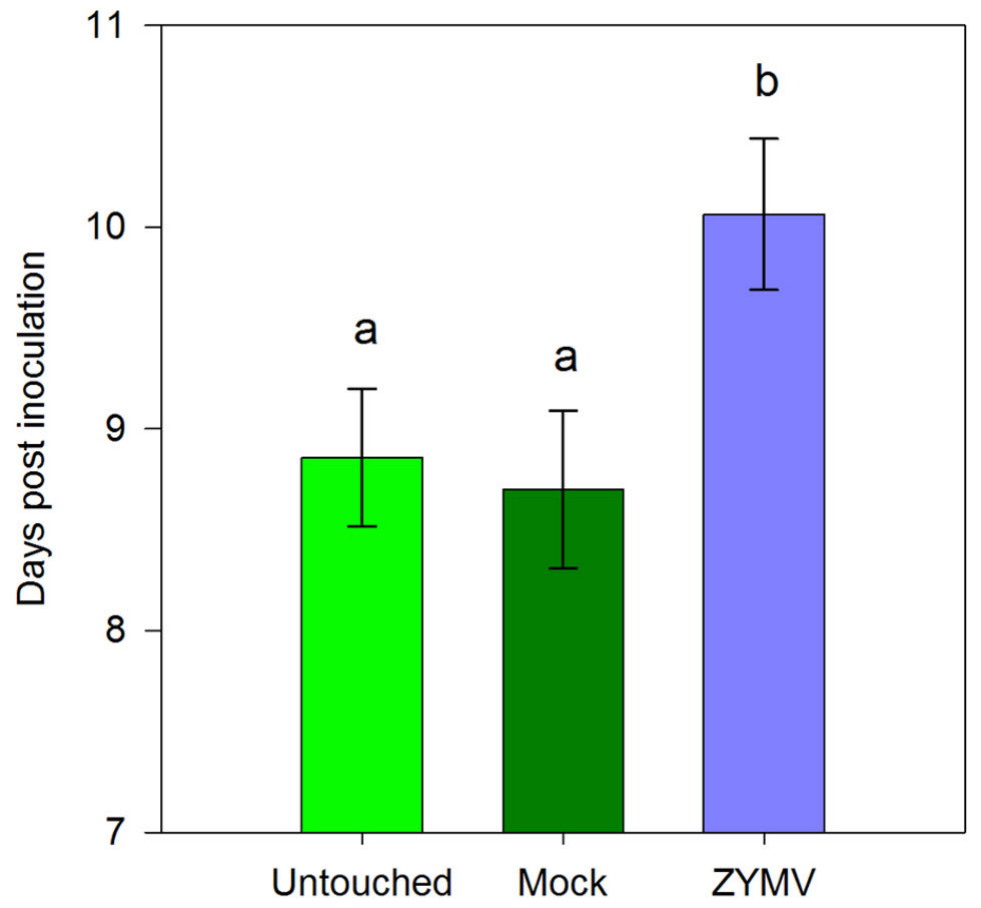
Timing of appearance of systemic wilt symptoms on plants within the high dose inoculation experiment. Bars represent mean day post inoculation of appearance of systemic symptoms (on a non-inoculated leaf) for untouched control (n = 49); mock (n = 50); and ZYMV-infected (n = 47) plants. Letters indicate significant differences (*P* < 0.05 by a Tukey-Kramer test).

In the inoculation experiment using infectious frass supplemented with *in vitro* bacteria, 99.32 % of inoculated plants developed wilt symptoms (all but one). There was no difference in the timing of symptom appearance on the first leaf (One-way ANOVA, *F*_2,143_ = 2.59, *P* > 0.05, Figure 5), though wilt symptoms spread to a second leaf on average one day later in ZYMV-infected plants compared to mock-inoculated plants or untouched controls (One-way ANOVA, *F*_2,143_=5.23, *P* = 0.0065, Figure 6). Whole-plant wilting also occurred more slowly in virus-infected plants compared to virus-free controls (One-way ANOVA, *F*_2,141_=6.11, *P* = 0.0029, Figure 5)

## Discussion

We consistently observed significant SA induction in ZYMV-infected plants, with even stronger SA responses in plants co-infected by ZYMV and *E. tracheiphila*, even though *E. tracheiphila* alone did not appear to elicit strong SA responses (Figure 2). We also observed a slight delay in the onset and progression of wilt disease symptoms in plants with prior ZYMV infections compared to mock inoculated controls at two different levels of exposure to the bacterial pathogen (Figures 4,5). Nevertheless, overall rates of *E. tracheiphila* infection success were not significantly influenced by the presence of ZYMV in co-infection. Thus, while our results are consistent with the possibility that ZYMV-induced SAR may make plants slightly less susceptible to *E. tracheiphila*, it seems unlikely that this effect could explain the significant reduction in wilt disease incidence previously observed for ZYMV-infected relative to uninfected plants in the field [3,4]. Therefore, the current study can be viewed as providing additional support for our hypothesis that the reduced incidence of wilt disease in virus-infected plants is explained primarily by reduced exposure to the wilt pathogen mediated by virus-induced effects on host-plant traits mediating interactions with the beetle vectors of the bacterial pathogen—including flower number and the olfactory cues presented to foraging beetles [3,5,7].

As noted, *E. tracheiphila* infection does not appear to significantly induce SA on otherwise healthy plants: no significant elevation was observed in *E. tracheiphila*-infected plants shortly after inoculation (Figure 2), nor five days later, by which time wilt symptoms had developed (Figure 3A). The interactions of bacteria with plant-defense signaling networks are highly variable across systems, but some bacterial pathogens appear to actively suppress host defenses by exploiting crosstalk between phytohormone signaling pathways. For example, some strains of *Pseudomonas syringiae* produce coronatine, a JA mimic that suppresses SA signaling [33]. The stronger induction of JA in response to *A. vittatum* feeding on *E. tracheiphila*-infected plants, relative to that observed on virus-infected plants or healthy controls, is intriguing in this regard, as the JA and SA pathways have been shown to interact antagonistically in some systems [24,25]. Bacterial genes, including the *avrE* genes of *P. syringae*, have previously been implicated in the direct suppression of host defenses [34]. The fire-blight pathogen *E. amylovora* contains an *avrE* homologue DspA/E (disease-specific region) that is required for pathogenicity on hosts [35,36]. This gene is also present in the *E. tracheiphila* genome [ETR_11262] (among other potential candidates, Shapiro et al. unpublished data) and might conceivably function as an SA-signaling suppressor for both *E. tracheiphila* and *E. amylovora*. 

Our results reveal no evidence of defense suppression or exploitation of SA-JA crosstalk by ZYMV. Treatments in which ZYMV was present in single infections or in co-infections with *E. tracheiphila* exhibited significantly higher levels of SA than uninfected control treatments (Figures 2,3A). Nevertheless, *A. vittatum* feeding induced JA responses in ZYMV-infected plants similar to those observed for healthy, mock-inoculated controls. This suggests that even relatively high levels of SA do not inhibit JA induction in response to herbivory in this system. However, we did previously observe an *A. vittatum* feeding preference for virus infected vs. healthy *C. pepo* ssp. *texana* [7] in a laboratory dual choice-feeding experiment—notwithstanding the reduced beetle attraction to virus-infected plants in the field documented in the same study—suggesting that ZYMV may otherwise alter plant nutritional and/or defense chemistry (or other plant traits) in ways that enhance the accessibility or quality of host plant tissues for the herbivore. 

Pathogen effects on host plant quality for vector and (less frequently examined non-vector) herbivores in fact appear quite common [37-44]. The apparently enhanced JA responses of *E. tracheiphila*-infected plants to subsequent feeding by *A. vittatum* (Figure 3B) are also interesting in this regard. JA mediates a broad suite of plant anti-herbivore defenses against chewing herbivores, likely including the increased production of cucurbitacins (toxic oxygenated triterpenes) in cucurbit plants. Cucurbitacins are among the most bitter compounds known—detectable by humans at levels of 1ppb—and effectively deter feeding by many herbivores. However, *A. vittatum*, and other Diabroticite cucumber beetles, are highly specialized and well adapted to the defenses of their host plants, including cucurbitacins, which indeed can act as feeding stimulants for these insects [45]. We previously reported *A. vittatum* feeding preferences for wilting vs. non-wilting *C. pepo* ssp. *texana* leaves [7], which likely reflect,, in part, the increased accessibility of wilting leaves, which exhibit reduced turgor pressure, as well as the effects of other gustatory and olfactory cues [7,46]. But JA-mediated enhancement of feeding-induced cucurbitacin production in *E. tracheiphila* infected plants could also enhance beetle preferences for infected tissues—from which they acquire the bacteria during feeding—with potential implications for the acquisition and spread of *E. tracheiphila* by beetle vectors. From the perspective of the pathogen, the transmission advantages of vector preferences for infected hosts must be weighed against the need for vectors to eventually disperse and carry the pathogen to uninfected individuals; however, in the case of bacterial wilt disease the extreme virulence of the pathogen (under field conditions) ensures beetle dispersal following the rapid death of the host plant. Thus, enhanced vector attraction to and feeding preferences for *E. tracheiphila*-infected hosts may well be expected to enhance transmission [7].

In conclusion, our findings suggest that prior infection by ZYMV slightly delays the subsequent onset and progression of bacterial wilt disease, but does not significantly reduce overall rates of infection success by *E. tracheiphila*. This observation supports the hypothesis that significantly reduced rates of wilt disease in virus-infected plants under field conditions are likely explained by reduced exposure to beetle vectors mediated by virus-induced changes in plant traits influencing plant-insect interactions [3,5,7]. We observed no clear evidence of antagonistic cross-talk between SA and JA signaling pathways in this pathosystem, but some evidence that *E. tracheiphila* successfully suppresses or evades the induction of SA-mediated defenses. *E. tracheiphila* furthermore appears to enhance the induction of JA in response to herbivory, which might, somewhat counter-intuitively, be expected to enhance host plant attractiveness to *A. vittatum* given the narrow specialization of this herbivore and its use of defensive cucurbitacins as feeding stimulants. Further work is needed to elucidate the detailed mechanisms of plant defense responses to co-infection by multiple pathogens and the implications of such interactions for broader patterns of disease ecology and epidemiology in this and other pathosystems.
